# Detection of the T1640C *RYR1* mutation indicating malignant hyperthermia in dogs

**DOI:** 10.17221/46/2023-VETMED

**Published:** 2023-11-29

**Authors:** Jana Haluskova, Beata Holeckova, Lenka Kokulova, Martina Galdikova, Jaroslav Bucan, Viera Schwarzbacherova, Silvia Sedlakova

**Affiliations:** Department of Biology and Physiology, University of Veterinary Medicine and Pharmacy in Košice, Košice, Slovak Republic

**Keywords:** allele, anaesthesia, disease, gene, sequencing

## Abstract

Malignant hyperthermia (MH) is a clinical syndrome exhibiting elevation of expired carbon dioxide, hyperthermia, muscle rigidity, rhabdomyolysis, acidosis and hyperkalaemia, as well as cardiac dysrhythmia and renal failure. The syndrome manifests itself as a response to anaesthetic agents, such as e.g., halothane, desflurane, and succinylcholine. Depending on the animal species, MH is characterised by autosomal dominant or recessive inheritance, and so far two genes have been identified whose mutations can be linked to MH: *RYR1* and *CACNA1S*. In different species, various mutations of the *RYR1* gene have been described which may underlie MH. One of these mutations in dogs is T1640C, which results in the substitution of alanine for valine of the amino acid 547 (V547A) in the RYR1 protein. In our work, we aimed to investigate MH at the DNA level by identifying the T1640C mutation in a group of 50 dogs. For this purpose we used the PCR-RFLP technique, and in six dogs also direct sequencing of PCR products and subsequent comparison of their sequences with the RYR1 gene sequence in an online database. The results of our study show that none of the dogs analysed had any mutant allele of the RYR1 gene, indicating that none should be affected by MH.

Malignant hyperthermia (MH) is a pharmacogenetic disorder characterised by a hyper-metabolic response to anaesthetic agents, such as halothane, desflurane, sevoflurane, and isoflurane or the depolarizing muscle relaxant succinylcholine. The clinical symptoms include an unexplained increase in expired carbon dioxide, hyperthermia, muscle rigidity, rhabdomyolysis, acidosis, and hyperkalaemia, as well as cardiac dysrhythmia and renal failure (Roberts et al. 2001; Rosenberg et al. 2015). It was found that the symptoms of MH are a consequence of uncontrolled release of intracellular Ca^2+^ from the sarcoplasmic reticulum (SR) in skeletal muscles, resulting in their abnormal metabolism (Sambuughin et al. 2005; Jiang et al. 2008). MH occurs not only in humans but also in animals, for example in pigs, but has also been observed in dogs and horses. In dogs (in contrast to humans), in addition to pharmacological triggers it is possible to induce an MH crisis through overexertion and stress (Mealey et al. 2019). The incidence of human MH events during anaesthesia is between 1 : 10 000 and 1 : 250 000 anaesthetics (Halliday 2003) and, on average, patients require three anaesthetics before triggering MH.

Depending on the species, MH is an autosomal dominant or recessive disorder, and so far two genes have been identified whose mutations can be definitely causally linked to MH: *RYR1* and *CACNA1S*. The *RYR1* gene codes for the ryanodine receptor, i.e. the Ca^2+^ channel in the SR membrane. All swine and up to 50% of human MH events appear to be associated with mutations in the *RYR1* gene (Roberts et al. 2001). According to Rosenberg et al. (2015), so far 50–70 percent of MH cases associated with more than 400 *RYR1* mutations have been identified. Lawal et al. (2020) found that during the last 30 years, 262 publications investigated MH and *RYR1*-related myopathies in preclinical model systems analysing more than 200 *RYR1* variations in a broad range of species including murine, porcine, avian, zebrafish, *C.* *elegans*, canine, equine and drosophila. Roberts et al. (2001) established a breeding dog colony through which they proved that MH in dogs is inherited in an autosomal dominant way. Moreover, they revealed that canine MH is caused by the T1640C *RYR1* mutation which gives rise to alanine for valine substitution of amino acid 547 (V547A) in the RYR1 protein, and that the MH susceptible (MHS) trait in the pedigree of mixed-breed dogs is in perfect co-segregation with this *RYR1* mutation.

Two laboratory *in* *vitro* contracture tests are currently used for the diagnosis of MH, which are based on muscle contraction after exposure to halothane or caffeine: the first test (IVCT) was developed by the European Malignant Hyperthermia Group (EMHG), and the second (CHCT) by the North American Malignant Hyperthermia Group (NAMHG) (The European Malignant Hyperpyrexia Group 1984; Larach et al. 1992). Alternative diagnosis of MH is available using methods based on DNA analysis including both the classical and next-generation sequencing of DNA isolated from muscle tissue biopsy samples. In the present work, we aimed to study MH at the DNA level by identifying the T1640C *RYR1* mutation in a group of 50 dogs coming from breeders based in the Slovak Republic. For this purpose we used the PCR-RFLP method, and in some dogs in addi tion to this method we identified the T1640C mutation based on sequencing of PCR products and comparison of their sequences with the *RYR1* gene sequence in an online database.

## MATERIAL AND METHODS

### Group of dogs and sample collection

Blood samples or buccal swabs were taken from a range of dogs within the Slovak Republic in the period 2020–2023 (Table 1). The above procedures complied with the national and institutional rules for working with animals (Decision of the Ethics Committee of the University of Veterinary Medicine and Pharmacy in Košice, Slovak Republic for the performance of procedures on animals in accordance with legislative requirements No. EKVP/2023-11). Peripheral blood was collected in sterile tubes with heparin or EDTA and buccal swaps were obtained swabbing the buccal mucosa with a special brush. The collected samples were stored in the freezer until DNA isolation.

**Table 1:** List and characterisation of the dogs screened for the T1640C *RYR1* mutation

Dog number	Breed	Gender	Age (years)	Sample type
1	Rhodesian Ridgeback 1	female	4	blood (EDTA)
2	Rhodesian Ridgeback 2	female	6	blood (heparin)
3	French Bulldog	female (positive PCR test for gender reversal)	0.5	blood (EDTA)
4	Bernese Mountain Dog	female	2	blood (EDTA)
5	Bavarian Mountain Scent Hound	female	1	blood (EDTA)
6	Hanoverian Scent Hound	female	2	blood (EDTA)
7	American Staffordshire Terrier	female	2.5	blood (EDTA)
8	Poodle	male	–	blood (EDTA)
9	Labrador Retriever	male	5	blood (EDTA)
10	Czechoslovakian Wolfdog	male	2	blood (EDTA)
11	Labrador Retriever	female	14	buccal swab
12	Jack-Russel Terrier	male	7	buccal swab
13	Border Collie	male	4	buccal swab
14	Long-Haired Collie (father)	male	6.5	buccal swab
15	Long-Haired Collie (mother)	female	9.5	buccal swab
16	Long-Haired Collie (progeny 1)	female	4	buccal swab
17	Long-Haired Collie (progeny 2)	female	4	buccal swab
18	Louisiana Leopard Dog 1	female	13	buccal swab
19	Louisiana Leopard Dog 2	female	10	buccal swab
20	Louisiana Leopard Dog 3	female	6	buccal swab
21	Louisiana Leopard Dog 4	male	6	buccal swab
22	Louisiana Leopard Dog 5	female	3	buccal swab
23	Shetland Sheepdog	female	9	buccal swab
24	Mongrel	female	15	blood (EDTA)
25	Border Collie	female	5	buccal swab
26	Shetland Sheepdog	male	10	buccal swab
27	Mongrel	female	5	blood (heparin)
28	Maltese Pinscher	male	2	blood (heparin)
29	Maltese Pinscher	female	8	blood (heparin)
30	Collie	female	6	buccal swab
31	Border Collie	male	2	buccal swab
32	Louisiana Leopard Dog 6	male	3	buccal swab
33	Louisiana Leopard Dog 7	male	8	buccal swab
34	Shetland Sheepdog	female	0.25	buccal swab
35	Border Collie	female	3	buccal swab
36	Australian Shepherd	male	2	buccal swab
37	Border Collie	male	5	buccal swab
38	Labrador Retriever	male	7	buccal swab
39	Australian Shepherd	male	5	buccal swab
40	Australian Shepherd	female	8	buccal swab
41	Miniature American Shepherd	female	1	buccal swab
42	Miniature American Shepherd	female	2	buccal swab
43	Slovak “hajčiarik”	female	1	blood/EDTA
44	Slovakian Chuvach	male	3	blood/EDTA
45	Slovakian Chuvach	male	3	blood/EDTA
46	Australian Shepherd	female	11	blood/EDTA
47	Australian Shepherd	female	7	blood/EDTA
48	Australian Shepherd	female	4	blood/EDTA
49	Australian Sheepdog	female	3	blood/EDTA
50	Australian Shepherd	male	5	blood/EDTA

Blood samples were taken at the Small Animal Clinic (University Veterinary Hospital, University of Veterinary Medicine and Pharmacy in Košice, Slovak Republic) and buccal swabs were obtained from breeders in Slovakia, both in the same period 2020–2023

### DNA isolation

DNA from the dog blood samples and buccal swabs was isolated using the ReliaPrep^TM^ Blood gDNA Miniprep System (Promega, Fitchburg, WI, USA) according to the manufacturer’s recommendations. The amount and purity of the DNA was evaluated using a TM P-class nanophotometer (IMPLEN, Munich, Germany).

### PCR-RFLP

To detect the T1640C *RYR1* mutation in the DNA of the analysed dogs, we first amplified a 487 bp stretch between exon 14 and 16 of the *RYR1* gene, and the amplicon was subsequently digested with enzyme *MlsI*. The PCR reactions were carried out in a volume of 25 μl, and the reaction mixtures contained the following reagents: 1 × concentrated GoTaq^®^ G2 Hot Start Polymerase reaction buffer (Promega, Fitchburg, WI, USA); 1.5 mM MgCl_2_ (Promega, Fitchburg, WI, USA); 0.2 mM dNTPs (Promega, Fitchburg, WI, USA); 0.25 μM of both the forward RYR1-14 and reverse RYR1-16 primer (Roberts et al. 2001) (Sigma-Aldrich, Saint Louis, MO, USA); 0.125 μl of the GoTaq^®^ G2 Hot Start Polymerase (5 U/μl; Promega, Fitchburg, WI, USA); nuclease-free water and about 10–20 ng of the dog template DNA. The amplification conditions were as follows: I/95 °C, 2 min; II/35 cycles: 95 °C, 40 s; 61 °C, 30 s; 72 °C, 1 min; III/72 °C, 5 minutes. The PCR amplification was performed on a Biometra Thermocycler (Analytik Jena, Jena, Germany). The subsequent cleavage reactions in a total volume of 20 μl contained the following reagents: 10 μl of PCR amplification mixture, 2 μl of 10 × concentrated reaction buffer for the *MlsI* restriction endonuclease (RE) (Thermo Fisher Scientific Baltics UAB, Vilnius, Lithuania), 1 μl of *MlsI* (Thermo Fisher Scientific Baltics UAB, Vilnius, Lithuania; 5 U/μl) and 8 μl of nuclease-free water. Plasmids pEX-A128-RYR1 MUT and pEX-A128-RYR1 WT were also cleaved with *MlsI,* and their cleavage mixtures with a total volume of 20 μl contained 5 μl of plasmid solution (approximately 10.7 ng/μl), 2 μl of 10 × concentrated reaction buffer for *MlsI* (Thermo Fisher Scientific Baltics UAB, Vilnius, Lithuania), l μl of *MlsI *(Thermo Fisher Scientific Baltics UAB, Vilnius, Lithuania; 5 U/μl), and 13 μl of nuclease-free water. The cleavage reactions were carried out at 37 °C for 4 h and they were stopped by incubation at 65 °C for 20 minutes. The PCR amplification products as well as the *MlsI* RE cleavage products were identified in the 2% agarose gel with the addition of the GelRed^®^ Nucleic Acid Stain (Biotium, Fremont, CA, USA). Both the GeneRuler 50 bp DNA ladder (Thermo Fisher Scientific Baltics UAB, Vilnius, Lithuania) and FastRuler^TM^ DNA ladder, Middle range (Fermentas, Waltham, MA, USA) were used as the molecular weight standards and the gels were photographed using a VWR GenoView transilluminator (Major Science, Taoyuan, Taiwan). The results of digestion were evaluated as follows: if a particular dog does not carry the mutant *RYR1* allele, only the 487 bp band should be present in its electrophoretic profile after digestion. In the case of a dog with only one mutant *RYR1* allele, bands of 287 and 219 bp resulting from digestion of the 487 bp band should be present in its PCR-RFLP profile in addition to the 487 bp band. If a dog has both mutant alleles, only the 286 and 219 bp bands should be present in its PCR-RFLP profile.

### Sequencing of PCR products and sequence alignment

Sequencing of PCR products was carried out commercially (Laboratory of Biomedical Microbiology and Immunology, University of Veterinary Medicine and Pharmacy in Košice, Slovak Republic) and for sequence comparison of PCR products of several dogs with the sequence of the *RYR1* gene in the online database (ENSEMBL), the Clustal Omega multiple sequence alignment program (https://www.ebi.ac.uk/Tools/msa/clustalo/) was used.

## RESULTS

**Figure 1:**
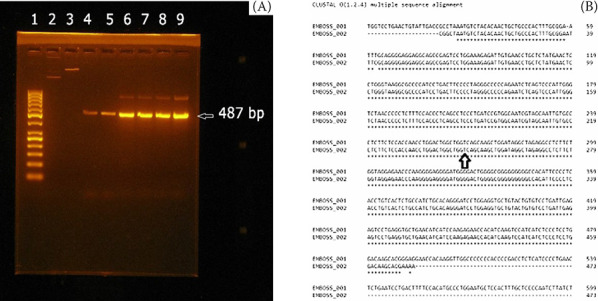
Detection of the T1640C *RYR1* mutation in DNA of dogs coming from breeders in the Slovak Republic (A) Electrophoretic analysis of PCR-RFLP profiles. Line 1: molecular weight marker (GeneRuler 50 bp DNA ladder; Thermo Fisher Scientific Baltics UAB, Vilnius, Lithuania). Line 2: plasmid pEX-A128-RYR1 WT digested with *MlsI*. Line 3: plasmid pEX-A128-RYR1 MUT digested with *MlsI*. Line 4–9: dog PCR products digested with *MlsI* (line 4 – dog 1; line 5 – dog 6; line 6 – dog 14; line 7 – dog 15; line 8 – dog 16; line 9 – dog 17). Arrow indicates the 487 bp band. (B) Alignment of the dog 15 PCR product sequence (bottom line) with the *RYR1* gene sequence using Clustal Omega software; arrow indicates tymin within the *RYR1* sequence which has not undergone the T1640C mutation, and stars indicate the consensual nucleotides

In our study, we found that in the PCR-RFLP profile of each of the 50 dogs analysed, only a 487 bp band and no smaller bands of 286 and 219 bp were present (Figure 1A).

In some dogs (e.g. 14–17), in addition to the 487 bp band, a larger band of about 650 bp was detected. We assume that this band may be the result of some differences in non-coding sequences in the *RYR1* exon 14–16 region between dog breeds. However, we did not take this band into account in our analysis. Together with the dog samples, we analysed the plasmids with the cloned *RYR1* fragments for control: the plasmid pEX-A128-RYR1 WT containing the *RYR1* fragment without the T1640C mutation and the plasmid pEX-A128-RYR1 MUT carrying the *RYR1* fragment with the mutation. After incubation with MlsI enzyme, as expected, the plasmid pEX-A128-RYR1 WT was not cleaved and the plasmid pEX-A128-RYR1 MUT was cleaved by *MlsI* (Figure 1A). In dogs number 1, 6, 14, 15, 16, and 17 we had the PCR amplicons sequenced in addition to digestion with the *MlsI* enzyme. The subsequent comparison of the amplicon sequences with the *RYR1* sequence from the ENSEMBL database using the Clustal Omega software revealed that no transition of T to C in the 1 640^th^ nucleotide of the *RYR1* gene occurred in any dog (Figure 1B). This was also consistent with the results of our PCR-RFLP analysis.

## DISCUSSION

Brunson et al. (1998) investigated a canine halothane/succinylcholine-challenged colony of Doberman-German Shepard-Collie dogs for the presence of the T1640C mutation. All dogs were also phenotyped by IVCT. More than 150 dogs without MH phenotype were screened for the mutation and the overall results showed that all dogs with a positive MH phenotype had the mutation, whereas all dogs with a negative MH phenotype lacked the mutation. In the present work, we found that in a group of randomly collected dogs from breeders in the Slovak Republic, none of them carried the T1640C mutation in the *RYR1* gene. Dogs 14–17 represent the parents and their two offspring, and if the T1640C mutation was present in the parents, it would be possible to investigate its inheritance. In the available literature, we did not find other studies focusing on the molecular-genetic analysis of MH, and moreover, those studying MH in a group of randomly collected dogs, as was the case in our study. Clearly, given the relatively rare occurrence of MH, a much larger number of samples as in our study would need to be analysed to determine the frequency of the T1640C *RYR1* mutation, also concerning its possible increased presence in some dog breeds. However, considering the severity of the symptoms of MH, it would be advisable to test for this T1640C mutation at least dogs that are likely to be put under general anaesthesia for either therapeutic or diagnostic reasons.

By means of our work, we have laid the foundations for such testing in the conditions of our laboratory. However, the use of heparin should be avoided in the future when taking blood samples from dogs. Although this did not cause us any particular problems in our work, it is generally known that heparin can inhibit PCR amplification (Satsangi et al. 1994; Yokota et al. 1999), so its use should be avoided.

Based on our results we could conclude that probably none of the dogs we analysed is likely to develop symptoms of MH after exposure to anaesthetics or due to stress.
